# Heart Rate and Heart Rate Variability in Healthy Preterm-Born Young Adults and Association with Vitamin D: A Wearable Device Assessment

**DOI:** 10.3390/jcm12247504

**Published:** 2023-12-05

**Authors:** Krista Björkman, Marita Valkama, Ella Bruun, Pauli Pätsi, Petri Kulmala, Mikko P. Tulppo, Markku Leskinen, Marja Ojaniemi

**Affiliations:** 1 Department of Pediatrics, Oulu University Hospital, Wellbeing Services County of North Ostrobothnia, Pohde, 90220 Oulu, Finland; 2 Research Unit of Clinical Medicine, University of Oulu, 90014 Oulu, Finland; 3 Medical Research Center, University of Oulu, Oulu University Hospital, Wellbeing Services County of North Ostrobothnia, 90014 Oulu, Finland; mikko.tulppo@oulu.fi; 4 Faculty of Medicine, University of Oulu, 90014 Oulu, Finland; 5 Research Unit of Biomedicine and Internal Medicine, University of Oulu, 90014 Oulu, Finland

**Keywords:** preterm birth, cardiac autonomic function, RMSSD, HRV, wearable device, Vitamin D status

## Abstract

Prematurity has been associated with impaired parasympathetic cardiac regulation later in life. Changes in heart rate (HR) and heart rate variability (HRV) may indicate a risk for future cardiac dysfunction. The putative role of Vitamin D on cardiac autonomic function in individuals born preterm (PT) remains unknown. This study involves monitoring autonomic cardiac regulation and Vitamin D concentrations in 30 PT and 16 full-term (FT) young adults in a free-living context. The PT subjects were born between 1994 and 1997 at Oulu University Hospital. The inclusion criteria were (1) being born ≤ 32 gestation weeks or (2) being born < 34 gestation weeks with a birth weight under 1500 g. Participants wore an Oura ring sleep tracer, a smart ring device, for 2 weeks to monitor cardiac autonomic function. Parameters related to autonomic cardiac regulation, lowest nighttime resting HR, and the root mean square of successive differences (RMSSD) to describe HRV were collected. PT males exhibited a tendency toward lower RMSSD (71.8 ± 22.6) compared to FT males (95.63 ± 29.0; *p* = 0.10). Female participants had a similar mean RMSSD in the FT and PT groups at 72.04 ± 33.2 and 74.0 ± 35.0, respectively. Serum 25-hydroxyvitamin D concentration did not correlate with cardiac autonomic function parameters. When assessing the lowest resting nighttime HRs and HRVs in a long-term, real-world context, healthy female PT young adults performed similarly to their FT peers. In contrast, the present study’s results suggest that male PT young adults exhibit impaired autonomic cardiac function, potentially putting them at risk for cardiovascular disease later in adulthood.

## 1. Introduction

One in ten infants in Europe are born prematurely [1]. Over the past few decades, the survival rates of extremely immature preterm (PT) infants have significantly improved, largely due to the widespread use of surfactant to treat respiratory distress syndrome (RDS). Advancements in life supporting medical device technology, development in support treatments including better nutrition, and overall progression of early care practices towards more gentle treatments have also contributed to better survival rates. According to previous studies [2,3,4,5,6], prematurity is associated with changes and abnormalities in cardiovascular function. These changes may have long-term consequences, as PT born children are susceptible to cardiovascular disease (CVD) also in adult life. PT born individuals are more likely to have elevated blood pressure, ischemic heart disease, changes in heart structure and function, but also impairment in the autonomic cardiac control [2,7].

The cardiovascular effects of premature birth are multifactorial, involving both prenatal and postnatal health, with nutrition playing a significant role [2,7]. The mechanisms of how prematurity is related to later cardiovascular morbidity are still poorly understood. During the last trimester of pregnancy, the fetal autonomic nervous system (ANS) is developing rapidly, and disruption of this sensitive developmental period by preterm birth may result in abnormalities related to stress responses. [8]. In addition, prolonged exposure to stressful procedures during neonatal care can contribute to altered programming of the brain, resulting in changes in the hormonal and autonomic regulation of the cardiovascular, and neural responses [8,9]. These early changes may last to later life, contributing to potential disturbances in ANS function [8].

A widely used indirect marker of the cardiac autonomic function is heart rate variability (HRV). It is noninvasive, relatively simple, and the measurements are easily repeatable. However, measurements during daily activities can be quite variable, thereby not providing accurate information on the overall cardiac autonomic health. Because of this, researchers have started to investigate and measure cardiac autonomic parameters such as heart rate (HR) and heart rate variability (HRV) during sleep [8]. Repeated HRV measurements during sleep can be used to assess cardiac autonomic function [8]. The measurements of HRV during sleep have been shown to provide a reliable assessment of RR intervals unaffected by internal or external factors [10].

HRV refers to the variation between heartbeats, with higher HRV values indicating better ANS activity [11]. PT born adolescents exhibit impaired autonomic cardiac function, as evidenced by slower HR recovery after maximal exercise and a lower resting HRV [12,13]. Studies have linked higher HRs and lower HRVs to impaired cardiac function and an increased risk of future cardiac autonomic dysfunction [14]. Altered sleep-time HRV may manifest many years before the clinical onset of CVD [15]. Commercial smart devices and wearable technologies that monitor HR and HRV [16] are now available for assessing cardiovascular fitness and recovery. One such device is the Oura sleep tracer (Oura Ring Generation 2, Oura Health Oy, Oulu, Finland; https://ouraring.com, accessed on 21 November 2019), a smart ring that simplifies cardiovascular monitoring by collecting physiological signals related to motion, HR, and HRV [17].

Preterm born individuals often require intensive and comprehensive support treatment in the neonatal intensive care units (NICUs) during the first days of life. Over the past few decades, the use of surfactant after birth has become increasingly common as well as the use of other medication and advanced medical devices. Previous research has shown that there might be an association with the treatments used during the intensive care period and the cardiovascular outcome [18,19]. Use of corticosteroids has been associated with impaired HRV and thus cardiac autonomic function [19].

Previous studies have also shown that Vitamin D (VitD) impacts cardiovascular health in both children and in adults [20,21,22,23,24,25,26], by influencing both physical performance and cardiac muscle contractility [20,26]. VitD may also play a role in cardiac autonomic function, as VitD deficiency has been shown to impair HRV in healthy adult subjects [27,28,29]. In addition, low VitD concentrations have been associated with a higher risk of CVD and increased overall mortality [20]. Still, the role of VitD in regulating cardiac autonomic function in the PT born population remains poorly understood.

While PT born adults may have compromised cardiac autonomic function, limited data are available as most studies have been conducted in experimental settings with short-term measurements. We hypothesized that cardiac autonomic function is impaired in healthy young PT born adults compared to their FT born peers, and that a low VitD level may be a contributing factor. The present study had two main aims: (1) to evaluate HR and HRV in a real-world setting among healthy young PT born adults using wearable technology and to compare these results to FT born controls and (2) to investigate the influence of VitD and physical activity on cardiac autonomic function in young PT born adults.

## 2. Materials and Methods

**Figure 1:**
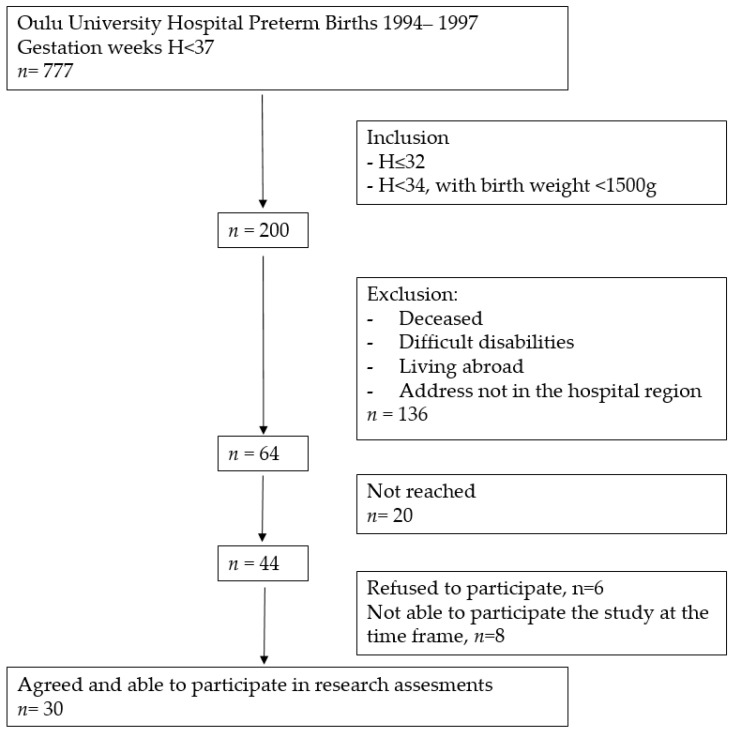
Flow chart of preterm born participants’ recruitment.

This research is part of a larger study in which the cardiovascular, musculoskeletal, and endocrinological health of young PT born adults and their peers are being investigated (Clinical Trial NCT04342078). PT born participants were born between 1994 and 1997 at Oulu University Hospital; they were eligible for the study if they were (1) born ≤ 32 gestation weeks or (2) born ≤ 34 gestation weeks with very low birth weight (VLBW, under 1500 g). During the 3 years 1994–1997 there were 777 premature births in Oulu University Hospital, Finland. Sixty-four eligible young adults met the inclusion criteria. Of these, 47% (*n* = 30) participated in the study. A more detailed flow chart of preterm group participants’ recruitment is presented in Figure 1.

To be able to compare the preterm born participants’ results with those born at full term (FT), age- and gender-matched healthy FT born peers (*n* = 16) were recruited from student and young adult populations living in the tertiary hospital district. Some of the full term born peers were recruited from voluntary medical and nursing students and their peers. Recruited controls were selected randomly from among the students. The participants did not receive any financial compensation for participating in the study but were offered comprehensive information on their own health gathered during the study protocol.

After the recruitment process, the final study group consisted of 46 young adults; 30 were born PT and 16 FT. Of the PT born participants, 14 (11 females, 3 males) were recruited before and 16 (8 females, 8 males) after the VitD intervention. In the same period, 16 peer control subjects who were born FT participated in the study. Information considering the neonatal period of the PT born adults was gathered from medical databases of the hospital. Birth data on the FT born peers were gathered by questionnaires.

The study participants were invited to the clinic for a study visit. The Oura ring sleep tracer, a multisensory wearable device, was provided to participants for use in this study; the ring size was carefully assessed to ensure the reliability of the measurements. This was carried out with the ring size assessment tool provided by the ring manufacturer. Participants received both verbal and written information on how to use the ring. They were guided to wear the ring at all times at least for 2.5 weeks, except during activities that could damage the device. The first 2 days of use were regarded as the habituation period and were not included in the final analyses. The smart ring measurements regarding HRV (RMSSD), HR, and MET-values were collected from Oura Cloud. Separate mean values and SDs for weekdays (Monday to Thursday) and weekend days (Friday to Sunday) were calculated to identify any possible differences between workdays and weekends. The mean values and SDs for the 2 weeks of measurements were calculated.

The parameters used in this study are as follows.

Heart rate (HR): The lowest resting nighttime HR; the HR is provided by the ring every 5 min when the user is sleeping.

Heart rate variability (HRV): The nighttime mean fluctuation between heartbeat intervals calculated by the Oura smart ring device using the root mean square of successive differences (RMSSD). The ring provides HRV values every 5 min when the user is sleeping.

Metabolic equivalent of task (MET): MET measures the rate at which a person expends energy relative to that person’s mass. The value indicates how many more calories are burned compared to resting consumption; 1.5 MET is the threshold for active calories.

During the visit to the clinic, the blood samples were drawn and the serum 25-hydrokxyvitamin D (S-25-OHD), plasma calcium (P-Ca), plasma phosphorus (P-Pi), and plasma alkaline phosphatase (AFOS) were measured. After the study visit, the investigator contacted the participant to inform them on the laboratory test results. VitD supplementation was started for the PT born participants when their VitD level was below 80 nmol/L to reach VitD levels between 80 and 120 nmol/L; levels were monitored once every 4 months. Serum 25-hydroxyvitamin D (S-25OHD) concentration was quantified by an immunochemiluminometric method at the Northern Finland Laboratory Centre (NordLab, Oulu, Finland). Calcium, phosphate, and alkaline phosphatase were quantified by a photometric method (NordLab, Oulu, Finland). All laboratory methods are accredited (SFS-EN ISO 15189:2013) by the Finnish Accreditation Service (FINAS, Helsinki, Finland).

Height and weight were measured from all study participants by the same investigator. Study height (cm, to an accuracy of 0.1 cm) was measured standing using a digital stadiometer (Seca gmbh & co. kg., Hamburg, Germany, mod: 2641900099). Study weight (kg, to an accuracy of 0.1 kg) was measured with light indoor clothing using digital flat scale (Seca Delta model 707, Seca gmbh & co. kg., Hamburg, Germany, mod: 3093309). Body mass index (BMI) was calculated as a person’s weight in kilograms divided by the square of height in meters.

Statistical analyses were performed using the Statistical Package for Social Sciences (SPSS, IBM Corp, Armonk, NY, USA) version 27.0. For continuous variables, independent sample *t*-tests were used. The results are given as mean values and SDs. Statistical significance is reported as the *p*-value for correlation between variables, with *p* < 0.05 considered significant.

## 3. Results

### 3.1. Study Participants

PT born participants were born at mean (SD) 30.3 (1.4) gestation weeks. The mean (SD) birth weight of PT born participants was 1361 (311) g; six (20%) had an extremely low birth weight (ELBW). The mean (SD) study ages were 24.4 (0.9) years in the PT group and 24.0 (1.0) in the FT group. Detailed characteristics of the participants are presented in Table 1.

### 3.2. Autonomic Heart Function Parameters

To investigate the putative association of prematurity with compromised cardiac autonomic function, HR and HRV were measured during nighttime periods over 2 weeks, and the results were compared to those of FT born young adults. There were no statistically significant differences in HR or HRV parameters between the PT and FT groups (see Table 2).

### 3.3. Gender Differences

As to differences between genders, FT born males had a significantly lower mean nighttime HR than all other participants (*p* = 0.01). This difference remained significant on both weekdays (*p* = 0.02) and weekend days (*p* = 0.01; see Table 2). PT born females had higher HR values than all other participants (*p* = 0.04). When comparing HR values between female and male PT born participants, the difference was statistically significant (53.1/min vs. 47.7/min; *p* = 0.03).

FT born males tended toward higher HRVs than FT born females (95.6 vs. 72.0; *p* = 0.20), while both PT born males and females had low HRVs (see Table 2). There was a tendency toward higher HRV values in FT born males when compared to PT born males (95.6 vs. 71.8; *p* = 0.10); this difference was not found between PT born females and FT born females (74.0 vs. 72.0; *p* = 0.88).

### 3.4. Association of Physical Activity with Cardiac Parameters

As physical activity has an impact on cardiac function, such activity was assessed with MET values. According to those results, PT born participants were as active as FT born participants (see Table 2). There were physically active participants exceeding resting consumption level (MET > 1.5) in both groups: five (16.7%) in the PT group and seven (43.8%) in the FT group (*p* = 0.08). These 12 physically active participants had higher nighttime HRV mean values than the 34 participants who had MET ≤ 1.5: 95.4 (48.3) vs. 68.3 (18.8); *p* = 0.08. A lower nighttime mean HR was also observed in the more physically active participants: 47.5 (7.5) vs. 51.5 (5.9); *p* = 0.07.

### 3.5. Association of Neonatal Factors with Cardiac Parameters

When evaluating the putative role of antenatal or postnatal dexamethasone treatment or neonatal characteristics (ELBW, SGA, RDS, and BPD), no association was found with the autonomic heart function variables HR, HRV, and MET in PT born participants.

### 3.6. Association of Vitamin D with Cardiac Parametres

To study the association of VitD concentration on cardiac autonomic function, S-25OHD levels were measured at the outset of recording cardiac function by a wearable device. Six (20%) participants in the PT group and four (25%) in the FT group had VitD insufficiency (S-25OHD levels under 50 nmol/L). FT born participants had lower S-25OHD levels than PT born participants (56.9 vs. 73.4; *p* = 0.02), of whom 28% (eight females and five males) received VitD supplementation to reach adequate VitD levels. None of the neonatal characteristics ELBW, SGA, RDS, or BPD was associated with S-25OHD levels; see Table 3 for full details.

S-25OHD concentration was not associated with HR (*p* = 0.47), HRV (*p* = 0.88), or MET (*p* = 0.66) values. When analyzing all participants together, the ten physically inactive participants (six PT and four FT) who had a mean MET ≤ 1.5 had low S-25OHD levels (under 50 nmol/L; *p* = 0.03).

## 4. Discussion

In the present study, we utilized an Oura smart ring as an ambulatory equipment to monitor long-term cardiac autonomic function in PT and FT born healthy young adults. In contrast to our hypothesis, no statistically significant difference was found in cardiac autonomic function parameters (HR, HRV) between the preterm and full term born groups of young adults at the age of 25. To our knowledge, this is the first study to assess autonomic cardiac regulation parameters in PT born young adults and to compare the results with their FT born peers in a long-term, free-living context. In our study, we assessed nighttime resting time-domain HRV over two weeks in healthy young adult PT born individuals to obtain accurate free-living average values for analysis. Earlier research showed that VLBW preterm children at age 13 had lower RMSSD values than their control peers when at rest [12]. One study of a non-disabled PT born population also suggested that adult parasympathetic regulatory capacity was significantly reduced in ELBW survivors at age 23 [30]. More recently, PT young adults exhibited RMSSD results of around 50 msec when recorded for 3–5 min in a seated position [31].

Prior studies have associated prematurity with poorer parasympathetic cardiac function. Our findings showed that the FT born males had the lowest nighttime HRs, while the PT born females had the highest nighttime HRs. This aligns with previous studies indicating a higher resting HR in females compared to males [32,33]. The lower HR in FT born males suggests better cardiac parasympathetic nervous system function than in PT born males, which is also supported by higher nighttime HRV values. For females, mean HR values did not significantly differ between the PT and FT born participants, suggesting that premature birth itself may not be a significant risk factor for compromised cardiac autonomic regulation, at least in this age group. However, it is important to note that our study only included healthy PT born participants and, thus, these results may not apply to PT born adults with existing health problems.

It is worth noting that nighttime RMSSD values are estimated to be 25–30% higher than daytime values for adults in the age range 20–30 [34]. In our study, nighttime resting HRV did not significantly differ between the young healthy PT and FT born females. However, there was a tendency toward lower HRV values in PT born males, indicating potentially poorer cardiac autonomic function, particularly during the weekends. Nonetheless, the difference in HRVs between male FT and PT born participants was not significant, likely due to the small sample size. Similar to our findings, previous research has reported significant HRV differences between genders [32,34,35,36].

Our study revealed that FT born participants in the present study were more physically active, with 43.8% of FT and 16.7% of PT born participants having MET values over 1.5 (*p* = 0.08). In a national cohort study of VLBW PT born subjects by Kaseva et al. (2015) [37], adult PT born participants achieved similar daily physical activity levels as their FT born peers. Our results suggest that physically active participants had lower HRs, indicative of good cardiac autonomic function and better recovery from daily living activities. In a previous study, healthy adults who were 10 years older than our cohort had mean MET (SD) values of 1.91 (0.14), as measured by ambulatory HRVs using an accelerometer for physical activity during waking hours [38]. Some reports also indicate that PT born adolescents and young adults do not recover as well as their peers from maximal exercise tests [39]. This finding is supported by their slower HR recovery and lower RMSSD values after equivalent physical activity [12,13].

Antenatal and postnatal variables from neonatal period had no association with cardiovascular parameters. The PT born young adults who had had RDS or BPD did not have impaired cardiovascular autonomic health evaluated with HRV and HR measurement results. Being born ELBW or SGA did not affect the cardiovascular autonomic parameters’ results in this study. In previous studies dexamethasone has affected preterm born children’s cardiac output responsiveness [18] and HRV on 14-year-old very low birth weight preterm (VLBW) adolescents [19]. However, in our study, the use of antenatal or postnatal corticosteroid did not affect the autonomic heart function variables.

In our study, the S-25OHD concentration in physically active participants was not associated with nighttime HRV. Both PT and FT born young healthy adults with average daily METs that stayed below 1.5 more frequently had S-25OHD levels below 50 nmol/L. Fourteen PT born participants entered the study before and sixteen after the VitD supplementation intervention study began, which may have influenced the difference in S-25OHD concentration between PT and FT born participants. Nevertheless, low VitD levels were common in both the PT and FT groups, with a quarter of the combined total exhibiting VitD insufficiency (S-25OHD < 50 nmol/L). This finding aligns with a national survey in which 21% of females and 26% of males aged 25–64 had a VitD insufficiency [40]. S-25OHD levels in our study were also comparable with those reported by Raulio et al. [40]. Our results underscore the importance of monitoring VitD levels in all young adults and considering active VitD supplementation.

Previous research presents conflicting results regarding the impact of VitD on cardiovascular health. Our findings align with a study involving a low cardiovascular risk population, where RMSSD values at age 28 did not differ between individuals with VitD deficiency and sufficiency [41]. However, another study by Mann et al. [28] reported that otherwise healthy 38-year-old subjects with VitD-deficient exhibited suppressed cardiac autonomic activity at rest. In a small intervention study with 33-year-old participants, cardiac autonomic response to stress tests improved when the mean baseline S-25OHD concentration, initially at 55 nmol/L, was increased to 100 nmol/L through VitD supplementation for 28 days [28]. This suggests that higher VitD levels may improve autonomic cardiovascular health.

Our study involved healthy young PT and FT born adults assessed at the same age. The Oura smart ring, known for its easy use and high validity in assessing nighttime HRs and time-domain HRVs, was used for data collection [17,42,43]. Recent research has found a low mean absolute percentage error in Oura ring HRV testing when compared to multi-lead electrocardiogram recordings [16]. Data collection with the smart ring spanned 2 weeks to minimize short-term ANS fluctuations related to stressful situations and daily changes. Recording began after a 2-day calibration period to prevent any ring adaptation bias. While physical activity measurements in PT born young adults are often based on questionnaires, introducing bias into the literature [44], our study employed an objective wearable device for physical activity data collection.

Given the limited number of participants, especially in the control group, the results of our study should be interpreted with caution. The amount of participants is low, as it is a part of a larger interventional study. The timeframe to use wearable device limited the entry to this study. It must also be noticed that there are several factors during the antenatal and postnatal period that affect the newborn health and cannot fully be taken into account when analyzing and interpreting these results. These and other factors affecting health during childhood and youth, such as use of corticosteroids and other medication, body structure, physical activity, and other ways of life, all have an impact on lifelong cardiovascular health in addition to being born prematurely. There might also be some common genetic factors predisposing both to premature birth and impaired cardiovascular autonomic function that cannot be taken into account in this study setting.

## 5. Conclusions

In summary, our study revealed subtle impairments in cardiac autonomic function in young male adults born preterm compared to their full-term counterparts. Vitamin D deficiency was prevalent in both groups but did not appear to impact heart rate and heart rate variability. It is important to note that our study participants were healthy, with no known cardiac symptoms or diagnoses. While these variations in cardiac autonomic function may suggest a risk for future cardiovascular disease, future research is needed to better understand potential associations as individuals age. In the future, wearable smart technology devices may serve as valuable tools for identifying preterm born adults at risk of cardiovascular disease later in life.

## Figures and Tables

**Table 1:** Characteristics of participants born preterm (PT) and full term (FT).

Clinical Values	PT (*n* = 30)	FT (*n* = 16)
Mean birth gestation weeks (SD)	30.3 (1.4)	39.7 (0.8)
Sex, Female/male (% male)	19/11 (37)	11/5 (31)
Mean birth weight, g (SD)		
All	1361 (311)	3520 (424)
Female	1357 (356)	3475 (472)
Male	1368 (230)	3620 (315)
ELBW, *n* (%)	6 (20)	-
SGA, *n* (%)	4 (13)	-
AND, *n* (%)	12 (40)	-
PND, *n* (%)	9 (30)	-
RDS, *n* (%)	20 (67)	-
BPD, *n* (%)	5 (17)	-
IVH gr II–III, *n* (%)	1 (3)	-
Age in years, mean (SD)		
All	24.4 (0.9)	24.0 (1.0)
Female	24.5 (1.2)	24.2 (0.9)
Male	24.0 (1.1)	23.7 (1.0)
Weight in kg, mean (SD)		
All	66.2 (10.4)	67.0 (11.0)
Female	63.8 (11.2)	63.9 (10.5)
Male	70.4 (7.7)	73.9 (9.4)
Height in cm, mean (SD)		
All	167.9 (8.0)	170.4 (7.9)
Female	163.7 (5.8)	167.0 (5.6)
Male	175.3 (5.8)	178.0 (7.1)
BMI, mean (SD)		
All	23.5 (3.4)	23.0 (3.2)
Female	23.8 (3.8)	22.9 (3.8)
Male	23.0 (2.4)	23.2 (1.0)
High school, *n* (%)	22 (73)	15 (94) *
University/College, *n* (%)	19 (63)	15 (94) *

FT = born full term; PT = born preterm; ELBW = extremely low birth weight; SGA = small for gestation age (birth weight and length below −2.5 SD); AND = antenatal dexamethasone; PND = postnatal dexamethasone; RDS = respiratory distress syndrome; BPD = bronchopulmonary dysplasia; IVH = intraventricular hemorrhage; BMI = body mass index. * Information missing for one participant.

**Table 2:** Autonomic cardiac function values in preterm (PT) and full-term (FT) groups.

Parameter	PT; *n* = 30 F/M 19/11	FT; *n* = 16 F/M 11/5	*p*-Value
Lowest Heart Rate (bpm): mean (SD)			
On weekdays			
All	51.1 (6.5)	49.0 (6.1)	0.29
Female	53.1 (6.1)	51.2 (5.5)	0.42
Male	47.7 (6.1)	44.0 (4.2)	0.24
On weekends			
All	51.9 (7.4)	48.4 (5.6)	0.10
Female	53.3 (7.0)	50.6 (4.9)	0.26
Male	49.3 (7.6)	43.5 (3.5)	0.13
Over two weeks			
All	51.4 (6.8)	48.7 (5.8)	0.18
Female	53.2 (6.4)	50.9 (5.2)	0.33
Male	48.4 (6.6)	43.8 (3.8)	0.17
RMSSD (ms): mean (SD)			
On weekdays			
All	73.6 (31.5)	78.0 (33.9)	0.66
Female	73.9 (36.2)	70.0 (33.6)	0.77
Male	73.0 (22.6)	95.6 (30.2)	0.12
On weekends			
All	72.8 (31.1)	81.3 (32.1)	0.39
Female	74.2 (35.1)	74.8 (33.1)	0.97
Male	70.3 (23.9)	95.6 (27.6)	0.08
Over 2 weeks			
All	73.2 (30.6)	79.4 (33.0)	0.53
Female	74.0 (35.0)	72.0 (33.2)	0.88
Male	71.8 (22.6)	95.6 (29.0)	0.10
Metabolic Equivalent Value: mean (SD)			
All	1.44 (0.2)	1.50 (0.2)	0.28
Female	1.44 (0.2)	1.50 (0.2)	0.40
Male	1.45 (0.1)	1.50 (0.2)	0.51

**Table 3:** Serum-25OHD concentration with related parameters in adults born preterm (PT) and full term (PT).

Laboratory Values	All PT; *n* = 30 F/M 19/11	FT; *n* = 16 F/M 11/5	*p*-Value
S-25OHD: nmol/L, mean (SD)			
All	73.4 (28.4)	56.9 (17.5)	0.02
Female	69.6 (24.1)	61.1 (16.7)	0.27
Male	80.0 (34.9)	47.8 (17.3)	0.07
Calcium: mmol/L, mean (SD)			
All	2.37 (0.08)	2.33 (0.08)	0.14
Female	2.35 (0.08)	2.34 (0.09)	0.82
Male	2.41 (0.06)	2.31 (0.05)	0.01
Phosphorous: mmol/L, mean (SD)			
All	1.14 (0.28)	1.23 (0.14)	0.23
Female	1.12 (0.33)	1.22 (0.13)	0.36
Male	1.17 (0.14)	1.24 (0.17)	0.35
Alkaline phosphatase: U/L, mean (SD)			
All	64.6 (13.1)	68.1 (25.1)	0.61
Female	59.2 (11.7)	65.5 (22.2)	0.32
Male	74.0 (9.8)	74.0 (32.8)	1.00

## Data Availability

The data presented in this study are available on request from the corresponding author. The data are not publicly available due to privacy.
